# Functional and Molecular Characterization of Rod-like Cells from Retinal Stem Cells Derived from the Adult Ciliary Epithelium

**DOI:** 10.1371/journal.pone.0033338

**Published:** 2012-03-14

**Authors:** Gian Carlo Demontis, Claudia Aruta, Antonella Comitato, Anna De Marzo, Valeria Marigo

**Affiliations:** 1 Department of Psychiatry, Neurobiology, Pharmacology and Biotechnology, University of Pisa, Pisa, Italy; 2 Department of Biomedical Sciences, University of Modena and Reggio Emilia, Modena, Italy; Telethon Institute of Genetics and Medicine, Italy

## Abstract

*In vitro* generation of photoreceptors from stem cells is of great interest for the development of regenerative medicine approaches for patients affected by retinal degeneration and for high throughput drug screens for these diseases. In this study, we show unprecedented high percentages of rod-fated cells from retinal stem cells of the adult ciliary epithelium. Molecular characterization of rod-like cells demonstrates that they lose ciliary epithelial characteristics but acquire photoreceptor features. Rod maturation was evaluated at two levels: gene expression and electrophysiological functionality. Here we present a strong correlation between phototransduction protein expression and functionality of the cells *in vitro*. We demonstrate that *in vitro* generated rod-like cells express cGMP-gated channels that are gated by endogenous cGMP. We also identified voltage-gated channels necessary for rod maturation and viability. This level of analysis for the first time provides evidence that adult retinal stem cells can generate highly homogeneous rod-fated cells.

## Introduction

Sight relies on specialized sensory neurons, retinal photoreceptors, which convert light stimuli into membrane potential changes necessary to transmit visual information to the central nervous system. Photoreceptors use an intracellular molecular cascade, called phototransduction, starting with the absorption of a photon by rhodopsin (Rho), which in turn activates the G protein transducin. The activated transducin binds to an inhibitory subunit of rod specific cGMP–phosphodiesterase (Pde6) increasing the rate of cGMP hydrolysis. The decrease in intracellular cGMP concentration then induces the closure of the cGMP-gated channels (Cng) at the cell membrane, and results in rod hyperpolarization.

Rod photoreceptors are intensely studied because they are implicated in some forms of retinal degeneration such as retinitis pigmentosa (RP). RP represents one of the most prevalent causes of visual handicap and is characterized by loss of rods, followed by cell death of cones, suggesting that rods are required to keep cones alive. Survival or transplantation of rods may thus provide a way to restore peripheral vision and prevent the loss of high-resolution central vision.

Several attempts to generate retinal neurons from embryonic stem (ES) cells and induced pluripotent stem (iPS) cells have been reported [1], [2], [3], [4], but they require long culture times and show low yield of rod-fated cells. Importantly, tissue specific stem cells have been identified at the marginal region of the adult retina and retinal stem cells can be derived and clonally cultured *in vitro* as retinal neurospheres (RNS) from the adult ciliary epithelium (CE) of several mammals [5], [6], [7]. Appropriate culture conditions can induce RNS to give rise to cells expressing some of the proteins typically present in rods. We previously showed that culture of RNS in the presence of bFGF and differentiation with medium containing serum, allow about 30–40% of the cells to express Rho and Pde6b [8]. The limitations of these studies were the generation of a mixed population of cells containing only a limited number of rods and the use of serum in the culture. Two reports also suggested that cells derived from the CE could not form retinal neurons [9], [10]. The lack of criteria for *in vitro* tracking rod development made difficult the evaluation of proper differentiation into rods.

By combining molecular and functional approaches we developed a differentiation protocol for RNS allowing a high percentage of cells to express several components of the phototransduction cascade. We demonstrate that cells *in vitro* not only express proteins typical of rod photoreceptors, but most importantly they generate cGMP, which opens cGMP-gated channels. Electrophysiological analysis highlighted that these cells do not reach full maturation *in vitro*, demonstrating the importance of functional approaches for assessing the efficacy of culture conditions in the development of cells with rod-like characteristics that may be applied for regenerative therapies and for drug testing-development.

## Results

### Differentiation of retinal neurospheres (RNS) into rod-like photoreceptor cells

**Figure 1:**
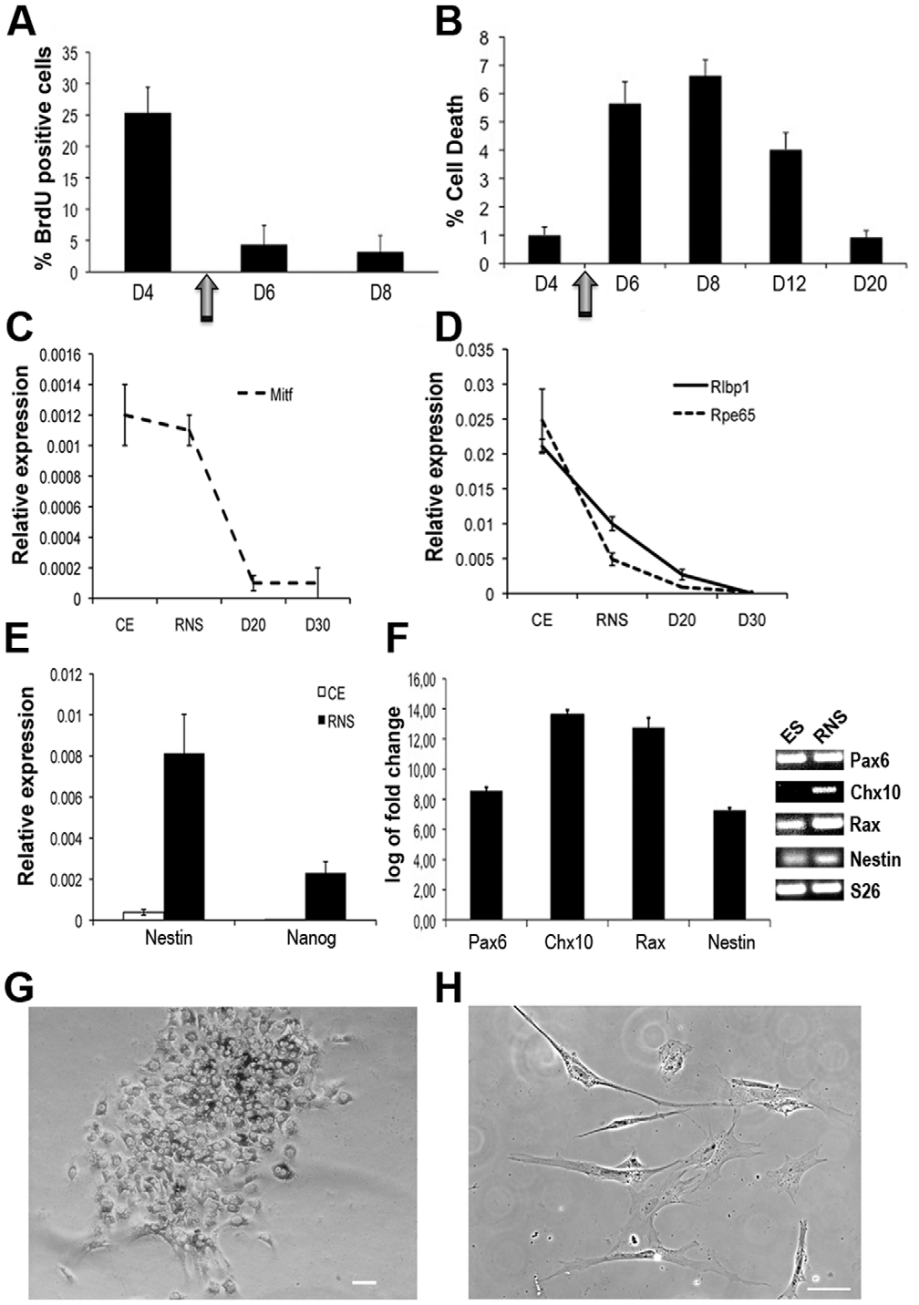
Differentiation of cells from RNS. (**A–B**) Changes in cells proliferation (BrdU) (A) or cell death (B) were analyzed at different times before and after treatment with differentiation medium at day 5 (indicated by an arrow). Data are derived from 8 fields from 2 independent experiments and represented as mean +/− s.e.m. (**C**–**D**) Real-time PCR analyses of CE markers *Mitf* (C), *Rpe65* (D) and *Rlbp1* (D; also expressed in Müller glia) show lower or similar levels in retinal neurospheres (RNS) compared to the ciliary epithelium (CE) and down-regulation of these genes during differentiation. (**E**) Real-time PCR analysis confirms higher levels of *Nestin* and *Nanog* mRNAs in RNS (black bars) compared to CE (white bars). In C, D and E data derive from the formula 2^−ΔCt^. *S26* was used as reference gene. (**F**) Real-time PCR analysis of retinal progenitors markers (*Pax6*, *Chx10*, *Rax* and *Nestin*) in RNS compared to expression in undifferentiated ES (set as 0). On the right-hand side of the graph the Real-time PCR products, after separation in an agarose gel, are shown. (**G–H**) Bright field images of cells at D4 (G) and D30 (H) show pigmentation reduction and changes in morphology in cells differentiated for 30 days. Scale bar is 50 µm.

We previously demonstrated that retinal progenitors, differentiated from RNS, acquire distinctive fates if exposed to different growth factors [8]. This underscored the importance of culture conditions for differentiation into specific cell types *in vitro*. We sought to enrich the population of cells acquiring a rod fate. To this purpose we seeded primary RNS onto an extracellular matrix substrate (see Methods) and allowed the cells to migrate out from the RNS and proliferate in the presence of bFGF. At the 5^th^ day (D5), cells were treated with differentiating medium containing factors known to favor rod differentiation: retinoic acid (RA) [11], sodium butyrate [12], T3 [13] and taurine [14]. Cells treated with differentiation medium quickly exited cell cycle, as demonstrated by reduced BrdU labeling (Figure 1A). Cells were induced to maturation, as suggested by gene expression (see below), and partially selected by the differentiation medium, as suggested by cell death observed during the first week of differentiation (Figure 1B).

**Figure 2:**
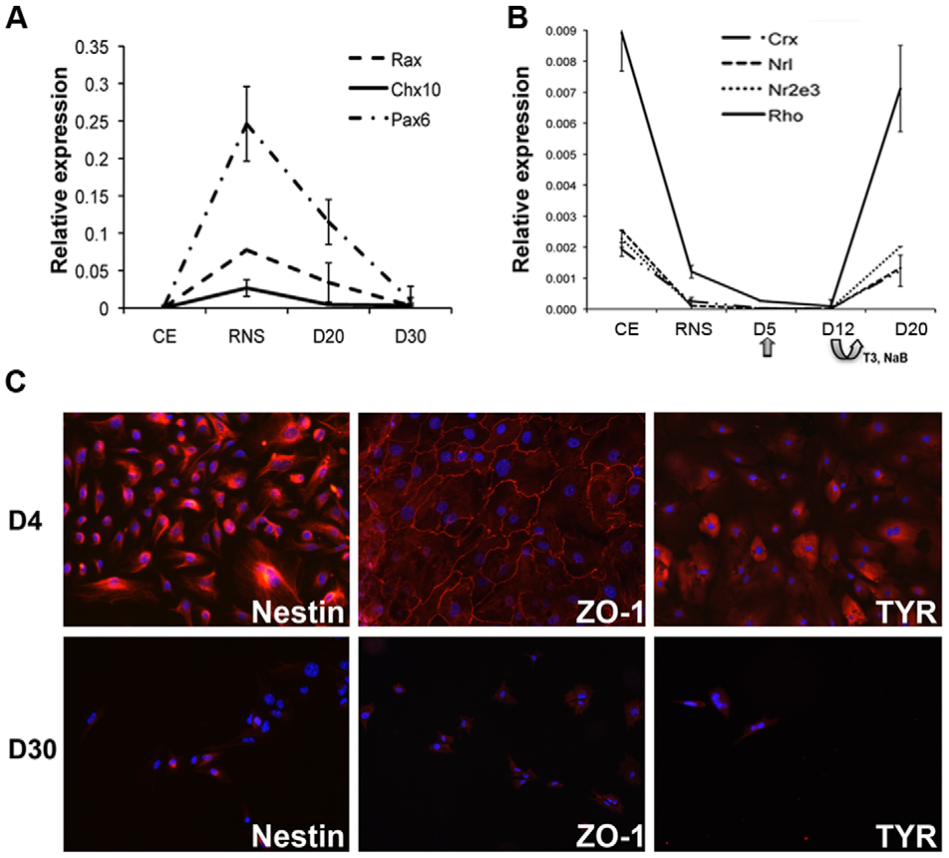
Expression analysis of RNS and differentiated cells compared to CE. (**A**) Real-time PCR analyses of retinal progenitor genes *Rax*, *Chx10* and *Pax6* show higher levels in retinal neurospheres (RNS) compared to the ciliary epithelium (CE) and down-regulation of these genes after 20 and 30 days of differentiation (D20-D30). (**B**) Time course analysis of mRNA levels of *Rho*, *Crx*, *Nrl* and *Nr2e3* in CE, RNS and cells at D5 (the day of exposure to differentiation medium, indicated by an arrow), D12 after 7 days in the presence of RA, taurine, T3 and sodium butyrate (bended arrow indicates withdrawal of T3 and sodium butyrate at this time point) and at D20 after removal of T3 and sodium butyrate. In A and B data derive from the formula 2^−ΔCt^. *S26* was used as reference gene. (**C**) Immunofluorescence experiments with antibodies detecting Nestin, ZO-1 and Tyrosinase (TYR) demonstrate the loss of expression of these proteins (red staining) upon differentiation. Upper row shows cells at D4 (before exposure to differentiation medium), lower row shows cells after 30 days of differentiation (D30). Blue shows nuclei labeled by DAPI.

Cells cultured with these conditions lost CE characteristics, as demonstrated by the down-regulation of genes such as *Mitf*, *Rpe65* and *Rlbp1* (Figure 1C–D) and the loss of epithelial morphology (Figure 1G–H). RNS also diverged from CE and were characterized by higher expression of stemness genes, such as *Nanog* (Figure 1E). We also observed higher mRNA levels of retinal progenitor markers (*Pax6*, *Chx10*, *Rax* and *Nestin*) in RNS compared to CE and to ES cells (Figures 1E–F and 2A). Differentiation caused progressive down-regulation of retinal progenitor markers (Figure 2A). Otherwise, expression of *Rho*, *Crx*, *Nrl* and *Nr2e3*, showing lower expression in RNS compared to the CE, was reactivated in cells after 12 days of differentiation (D12 in Figure 2B), when T3 and sodium butyrate were withdrawn from the culture (see Methods). RNS-derived cells did not re-acquire CE characteristics: Nestin, the tight-junction epithelial protein ZO-1 and the melanin synthesis enzyme Tyrosinase, while present in cells at D4, were not detectable in cells at D30 (Figure 2C).

**Figure 3:**
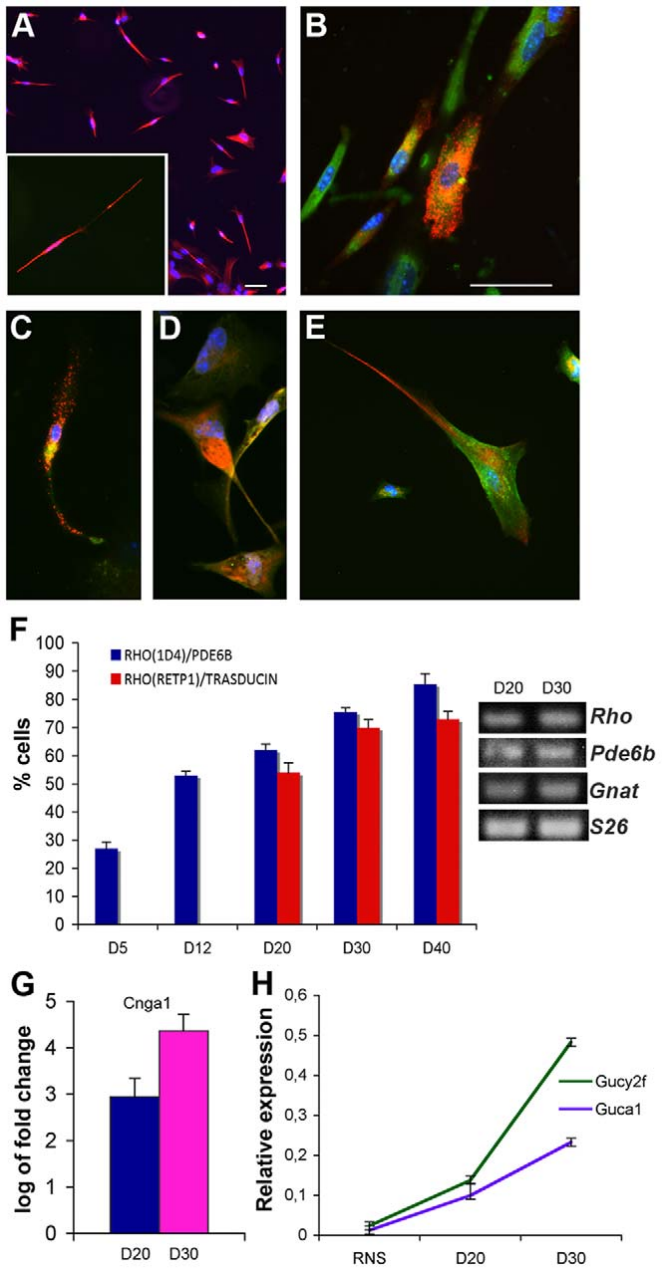
Expression of components of the phototransduction cascade in RNS-derived rods. RNS-derived rods were analyzed after 40 days of differentiation. Rho protein at the cell surface was detected with two different monoclonal antibodies: 1D4 (**A**) and RET-P1 (**B**). The magnification in A shows an example of a Rho^+^ cell labeled with 1D4 recognizing an intracellular epitope and requiring permeabilization of the sample. The antibody recognizing an extracellular epitope (RET-P1) and therefore detecting the protein at the cell surface (B) is characterized by a different labeling compared to the staining with 1D4 (A). (**B**) Double-labeling with antibodies for Rho (red) and Transducin (green). (**C**) Double-labeling with antibodies for Rho (red, RET-P1) and Recoverin (green). (**D**) Double-labeling with antibodies for Rho (red, 1D4) and peripherin (green). (**E**) Double-labeling with antibodies for Rho (red, 1D4) and Pde6b (green). Scale bars are 50 µm. Scale bar for C, D and E are equal to that shown in B. (**F**) Time course experiment analyzing RNS-derived cells co-expressing either Rho (1D4)+Pde6b (blue bars) or Rho (RET-P1)+Transducin (red bars) at different times of differentiation *in vitro*. Data are represented as mean +/− s.e.m. and derived from 10 fields from 2 independent experiments. On the right-hand side we show mRNA for *Rho*, *Pde6b*, *Gnat1* (transducin) and *S26* detected by RT-PCR at D20 and D30 confirming the expression these genes. (**G**) Real-time PCR for *Cnga1* mRNA shows an increase in RNS-derived cells after 20 (D20) and 30 (D30) days of differentiation compared to expression in RNS (set as 0). (**H**) Real-time PCR for *Gucy2f* and *Guca1* show up-regulation of these genes during differentiation. Data derive from the formula 2^−ΔCt^.

Rho expression was analyzed with two different antibodies that labeled similar numbers of cells and more than 90% of cells were Rho^+^ after D30 (Figure 3A–B). We calculated the number of cells co-expressing at least 2 markers of the phototransduction cascade: Rho and transducin, Rho and Pde6b, Rho and peripherin, Rho and recoverin (Figure 3B–E). While Pde6b was detectable at D5, we detected transducin only after D20 (Figure 3F). We observed that the numbers of cells co-expressing two markers of the phototransduction cascade increased over time in culture and were similar after 30 days *in vitro* (Figure 3F). mRNA levels of cGMP-gated channel (*Cnga1*), guanylate cyclase (*Gucy2f*) and its activator *Guca1a* (Guanylate cyclase-activating protein 1) also increased upon *in vitro* differentiation (Figure 3G–H).

Finally, we analyzed expression of proteins that are not photoreceptor specific but are expressed in other retinal cell types. Compared to our previous studies in the presence of serum [8], we found sporadic cells with bipolar characteristics expressing Pkcα and Goα, with amacrine characteristics expressing syntaxin, and with horizontal characteristics expressing calbindin (data not shown).

### Functional evaluation of cGMP-gated channels and cGMP turnover in RNS-derived rods

We undertook a functional analysis of rod-like cells derived from RNS evaluating, by patch-clamp analysis, the functional impact of genes coding for cGMP-gated channels and cGMP synthesis and degradation pathways (see Figure 3).

**Figure 4:**
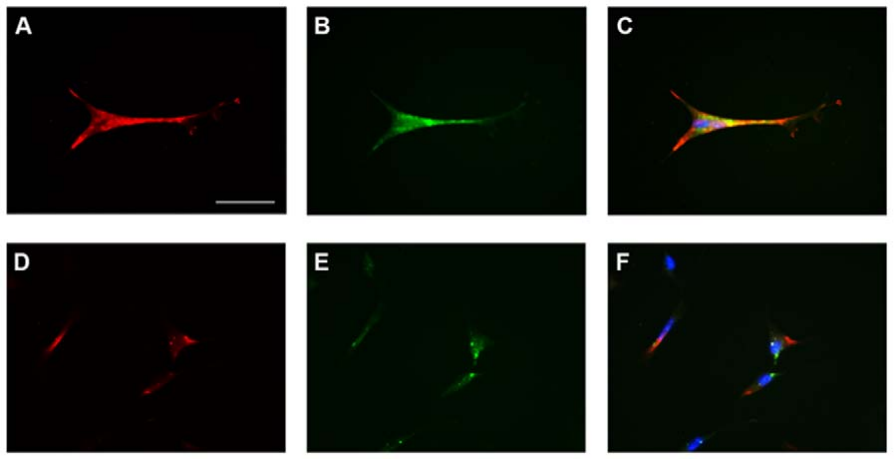
RNS-derived cells infected with AAV2/8-pRho-EGFP. RNS-derived cells were infected with AAV2/8-pRho-EGFP 4 days after seeding on ECM and analyzed after 30 days of differentiation. Expression of EGFP was evaluated at the fluorescence microscope (green in B, C, E and F) and co-expression of rod specific proteins was evaluated by immunolabeling with anti-Transducin (red in A and C) and anti-Rhodopsin antibodies (red in D and F). Blue is DAPI staining of nuclei in merged images (C and F).

In order to identify rod-like cells from those that had not undertaken this differentiation program, we genetically labeled the cells with the AAV2/8 viral system expressing EGFP under the control of the Rhodopsin promoter (AAV2/8-pRho-EGFP) [15]. At D4 cells were exposed to AAV2/8-pRho-EGFP, one day before treatment with differentiating medium that induced cells to exit the cell cycle. This protocol prevented AAV genomes dilution due to cell proliferation and allowed EGFP labeling of most of cells expressing Rhodopsin (Figure 4).

**Figure 5:**
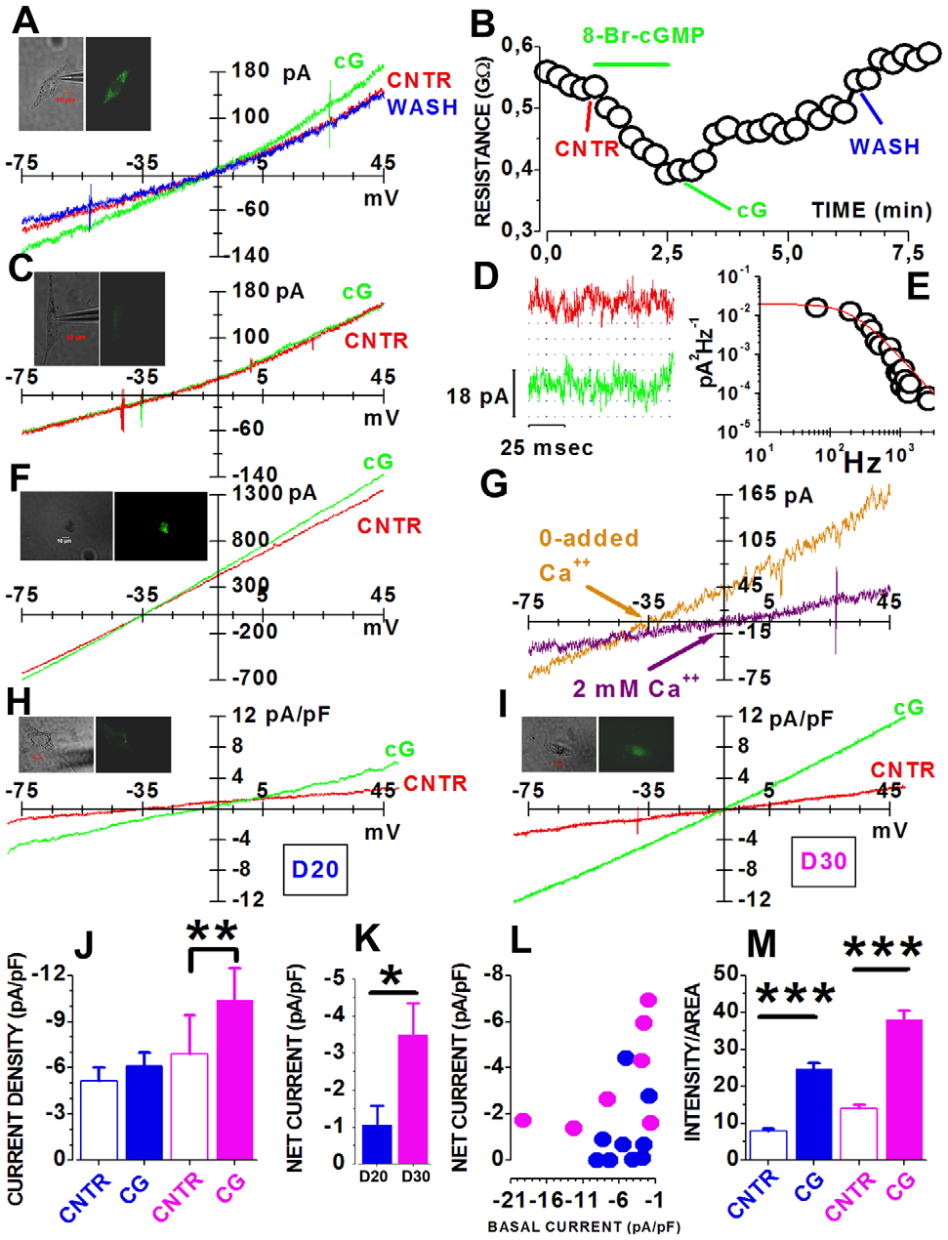
cGMP-gated currents in RNS-derived rod-like photoreceptors. (**A**) I/V relations before (red, CNTR), during (green, cG) and after (blue, WASH) application of 25 µM 8-Br-cGMP. (**B**) Membrane resistance changes during the 90 s-long 8-Br-cGMP application (green bar). CNTR, cG and WASH mark the time of acquisition of sweeps plotted in A. (**C**) I/V curves from a cell with low fluorescence (see Methods) before (red, CNTR) or during (green, cG) exposure to 25 µM 8-Br-cGMP. Each trace is the average of 4 sweeps. (**D**) 100 ms-long current stretches recorded before (red) or during (green) application of 25 µM 8-Br-cGMP. Dotted blue lines were drawn at 6 pA intervals. (**E**) Power spectrum (circles) of 8-Br-cGMP-induced current (see Methods) with the best fit by Eq. 3 (red line). (**F**) I/V in Locke's saline with 0-added Ca^2+^ before (red) or during (green) application of 100 µM 8-Br-cGMP. (**G**) Net currents activated by the cGMP analogue for cells in A (purple) and F (orange). Purple and orange arrows indicate reversal potentials in 2 mM (N = 7) and in 0-added Ca^2+^ (N = 3), respectively. (**H** and **I**) I/V curves before (red sweep) and during (green trace) application of 20 µM 8-Br-cGMP in a D20 (H) and in a D30 (I) RNS-derived rod. Current densities were computed using capacitances of 35 (H) and 29 pF (I). Vertical deflections in A, C, F, H and I are perfusion artifacts. (**J**) Average current densities at −80 mV for D20 (blue, N = 9) and D30 (purple, N = 7) RNS-derived cells, before (open bars) and during (filled bars) application of the cGMP analogue. Average capacitances were 25.2±4.9 pF and 38.98±7.21 pF at D20 and D30. (**K**) Average net current densities at D20 (blue, N = 7) and D30 (magenta, N = 9). (**L**) Net current densities as a function of basal current (before 8-Br-cGMP application) at −80 mV for D30 (magenta) and D20 (blue) cells. (**M**) Average fluorescence intensity per area of Ca^2+^ accumulation for D20 (blue) and D30 (purple) cells incubated in the absence (open bars, D20 N = 33; D30 N = 20) or in the presence (filled bars, D20 N = 21; D30 N = 19) of 20 µM 8-Br-cGMP. *, P<0.05; **, P<0.01; ***, P<0.001, Tukey's tests after two-way ANOVA. In A, C, F, H, I images of recorded cell are above records.

**Figure 6:**
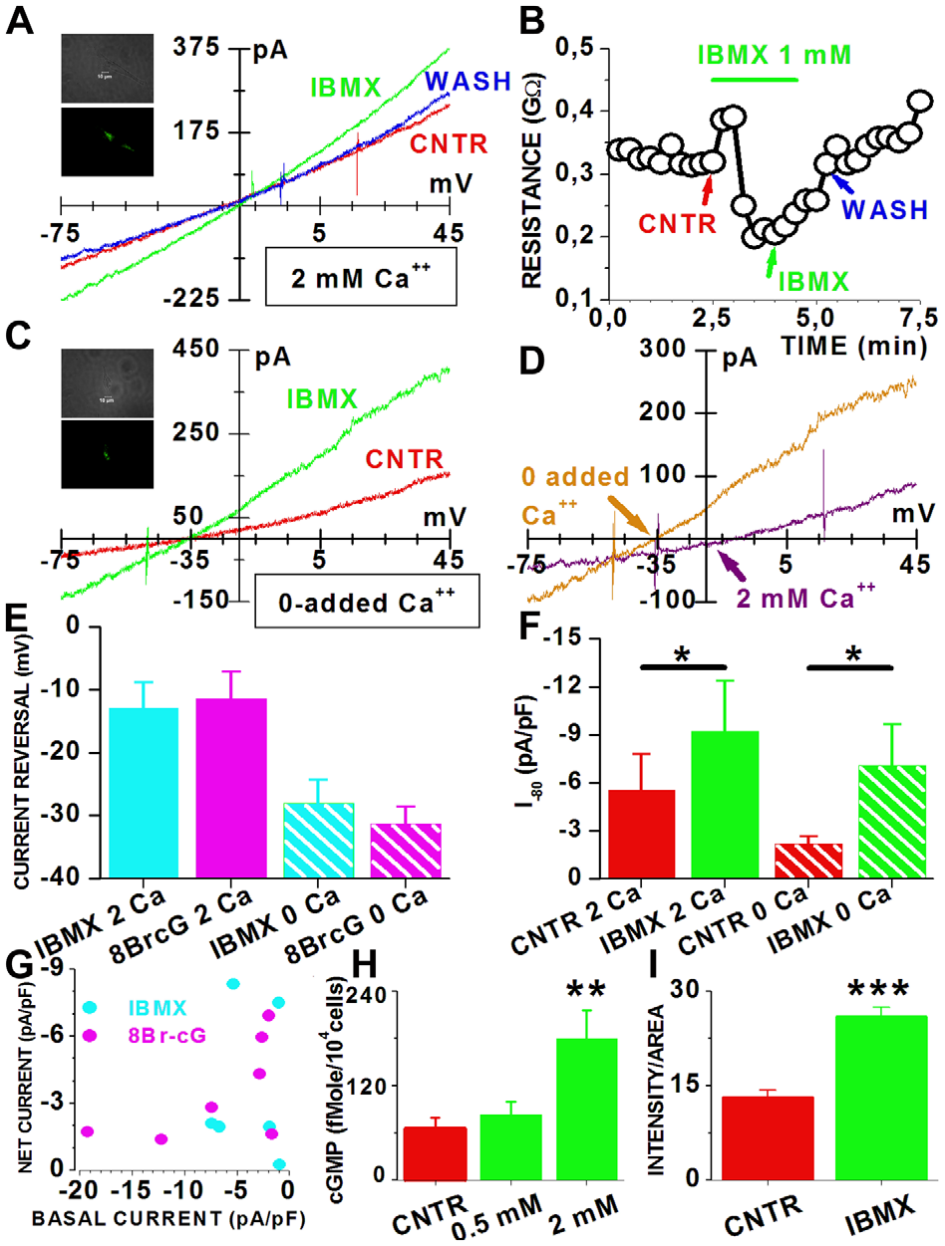
IBMX-gated currents in RNS-derived rod-like photoreceptors. (**A**) I/V relations for a D30 cell before (red, CNTR), during (green, IBMX) and after (blue, WASH) perfusion with 1 mM IBMX. (**B**) Membrane resistance changes during the 120s-long IBMX application (green bar). CNTR, IBMX and WASH mark the time of acquisition of sweeps plotted in A. The perfusion line was preloaded with IBMX. The initial increase in membrane resistance with IBMX was a perfusion artifact. (**C**) I/V relations for a D30 cell before (red, CNTR) and during (green, IBMX) perfusion with 1 mM IBMX in 0-added Ca^2+^. (**D**) Net IBMX-activated currents, computed from traces in A (purple) and C (orange). Reversal potentials are indicated by the purple and orange arrows. (**E**) Average reversal potentials in 2 mM Ca^2+^ (filled bars) or in 0-added Ca^2+^ (striped bars) of IBMX-induced (blue) and 8-Br-cGMP (magenta) currents. The effect of calcium on reversal potential was significant (P<0.001 by two-way ANOVA). (**F**) Average current densities before (red) and during (green) 1 mM IBMX application, in 2 mM (filled bars, N = 6) or 0-added Ca^2+^ (striped bars, N = 3). (**G**) Net current densities as a function of normalized basal current of D30 cells in response to 8-Br-cGMP (magenta) or IBMX (blue). (**H**) Average cGMP accumulation by D30 cells, in the absence (red - CNTR) and in the presence of 0.5 and 2 mM IBMX (green). The difference between control and 2 mM IBMX was significant. (**I**) Average calcium accumulation in control medium (red – CNTR, N = 20) and in 2 mM IBMX (green – IBMX, N = 29). *, P<0.05; **, P<0.01; ***, P<0.001, by one-way ANOVA followed by Tukey's tests. In A and C images of recorded cell are above records.

To evaluate the functional significance of *Cnga1* expression by RNS-derived rods, we measured cGMP-gated currents using the patch-clamp technique. *Cnga1* is a member of the superfamily of potassium channel genes coding for the pore-forming subunit of rod-type cGMP-gated channels [16]. In mammals, the main biophysical features of cGMP-gated channels are low unitary conductance [17], lack of voltage-dependence and reversal of current flowing through the channels close to 0 mV, due to their permeability to Na^+^, K^+^ and Ca^2+^ ions [18]. Data in Figure 5A show the reversible increase in membrane current induced in an EGFP-positive cell by the application of 8-Br-cGMP, a membrane-permeant analogue of cGMP. Properties of the current induced by 8-Br-cGMP were consistent with the opening of cGMP-gated channels in three ways. First, the cGMP analogue did not appreciably increase current noise, consistent with the opening of channels having low unitary conductance, as shown in Figure 5D that plots a 100 ms-long current stretch at an higher amplification than in Figure 5A, to show that 8-Br-cGMP (green traces) did not visibly increase current fluctuations compared to control records (red trace), as expected from the gating of channels of small unitary amplitude and brief open time. From the 8-Br-cGMP-induced variance (see Methods), we estimated a unitary event amplitude close to −0.2 pA at −80 mV. From power spectra of current fluctuations before and during exposure to the analogue we computed the difference spectrum, whose cut-off corresponded to a time constant of 0.75 ms, in reasonable agreement with the value of about 0.5 ms reported for native channels [19] (Figure 5E). Second, the time-course of cGMP effect on membrane resistance closely followed the equilibration time of the superfusing solutions (1–2 minutes), indicating that channels opened rapidly in response to the analogue (Figure 5B). Complete recovery occurred shortly after switching back to control saline without cGMP analogue (Figure 5B), consistent with gating not involving a slowly-reversing phosphorylation step. Accordingly, similar responses to 8-Br-cGMP were also measured from cells recorded in whole-cell mode with no ATP and GTP in the pipette (not shown), indicating that a phosphorylation step was dispensable for 8-Br-cGMP responsiveness. Third, the net current activated by the cGMP analogue reversed close to −10 mV (Figure 5G), a value consistent with the mixed cationic permeability of rod cGMP-gated channels, and the linear relation between current and voltage was consistent with the lack of strong voltage-dependent rectification. We also tested 8-Br-cGMP in cells primed in saline with no-added Ca^2+^ to reduce the electrochemical gradient for calcium (Figure 5F). In these conditions the cGMP-induced current reversed at more negative potentials than in the presence of 2 mM Ca^2+^. A comparison plot of the net 8-Br-cGMP-induced currents between cells in panels A and F is shown in Figure 5G. The reversal potential was close to −10 mV in 2 mM extracellular Ca^2+^ (purple arrow) and to about −30 mV in the absence of added Ca^2+^ (orange arrow), as expected for calcium-permeable channels. The average reversal potentials were −11.38±4.29 mV in the presence of 2 mM extracellular Ca^2+^ and −31.24±2.78 mV in the absence of added Ca^2+^ (see also Figure 6E).

In control experiments, cells with either no or faint EGFP expression did not respond to the cGMP analogue, suggesting that cGMP-gated channels were selectively expressed by cells committed to a rod fate, as shown in Figure 5C for a cell with low EGFP expression. Similar currents in response to 8-Br-cGMP were recorded from cells treated according to the differentiation protocol but not transformed with AAV2/8-pRho-EGFP (data not shown), ruling out possible unspecific effects of either EGFP or viral infection.

We, then, investigated the time-course of cGMP-gated channels expression by comparing current amplitudes at D20 and D30 to assess their consistence with *Cnga1* expression data (see Figure 3G). To avoid the confounding factor represented by the variability in cell sizes and morphologies (see Figure 5A, C, F, H and I), we evaluated developmental changes in cGMP-gated channels expression using current densities, obtained by normalizing current amplitudes to the cell electrical capacitance (an index of membrane area), rather than using raw current amplitudes. The I/V slope in response to 8-Br-cGMP was steeper at D30 (green in Figure 5I) than at D20 (green in Figure 5H), indicating that cGMP-gated channels density increased during maturation *in vitro*. Average normalized current amplitudes showed that cGMP-gated currents (magenta filled bar in Figure 5J) were significantly increased over control (magenta open bar) at D30. On the other end, at D20 8-Br-cGMP (blue filled bar in Figure 5J) did not significantly increase current density over control (blue open bar). The net increase in normalized current amplitudes induced by the cGMP analogue were also significantly larger at D30 than at D20, confirming the up-regulation of *Cnga1* observed at the mRNA level (Figure 5K).

Differentiation into rod-like cells affected the amplitude of currents measured in the absence of cGMP (open bars in Figure 5J). This increase in basal current with time, although not statistically significant, might indicate that endogenous cGMP was gating a fraction of channels. Datapoints in Figure 5L plot the net current increase induced by the cGMP analogue as a function of normalized current amplitudes measured before 8-Br-cGMP application. Cells with a fraction of their cGMP-gated channels already activated by endogenous cGMP were expected to have large inward basal currents, but small 8-Br-cGMP-induced currents. At D30 2 out of 7 cells had large inward basal currents and small 8-Br-cGMP-induced currents (Figure 5L, magenta filled circles), suggesting that in a fraction of D30 cells cGMP-gated channels were activated by endogenous cGMP. On the other hand, 7 out of 9 cells at D20 (blue filled circles) had either small currents or did not respond at all to the cGMP analogue, suggesting that the expression of cGMP-gated channels started at around D20. However, in the absence of specific inhibitors of cGMP-gated channels, we could not rule out that the increase in basal current resulted from an increased density of cGMP-independent channels. To address this point we measured relative calcium accumulation by rods in the presence or in the absence of 8-Br-cGMP at D20 and D30, exploiting the calcium permeability of cGMP-gated channels. Consistent with patch-clamp data, treatment with the cGMP analogue increased calcium ion accumulation inside the cells (filled bars in Figure 5M). Calcium accumulation in the absence of the cGMP analogue (open bars in Figure 5M) also increased with time of differentiation and the effect was statistically significant, suggesting that the number of channels gated by endogenous cGMP increased during rod maturation.

Although data described so far may indicate that a subset of D30 RNS-derived cells generate enough cGMP to activate a fraction of their cGMP-gated channels, it is unclear whether their cGMP levels were balanced through phosphodiesterase (Pde) degradation. To address this point, we recorded membrane currents from cells exposed to a Pde inhibitor. A reversible increase in membrane current was induced by the application of 1 mM of the Pde inhibitor Iso-Butyl-methylxanthine (IBMX) [20]) in an EGFP-expressing cell (Figure 6A). Similar to 8-Br-cGMP, the effect of IBMX was rapidly reversible upon washout (Figure 6B). In order to compare IBMX-activated currents with those measured in response to 8Br-cGMP we tested the effects of reducing the calcium electrochemical gradient. In the absence of added Ca^2+^, IBMX was still able to induce an increase in membrane current (Figure 6C), consistent with the activation of channels having mixed cationic permeability. The comparison between net currents induced by IBMX in 2 mM extracellular Ca^2+^ and no added Ca^2+^ showed differences in current reversals that were about −15 mV (Figure 6D, purple arrow) and −35 mV (Figure 6D, orange arrow) in 2 mM Ca^2+^ and 0-added Ca^2+^, respectively. The similarity of these results with those obtained with 8-Br-cGMP and IBMX was also evident when we plotted average values for the reversal potential of currents induced by IBMX (Figure 6E, blue bar) and 8-Br-cGMP (Figure 6E, magenta bar) in the presence of 2 mM extracellular Ca^2+^ (filled bars) and in the presence of reduced extracellular Ca^2+^ (striped bars). Two-way ANOVA test indicated a significant difference between 2 mM and 0-added Ca^2+^, but no significant difference between 8-Br-cGMP and IBMX.

We then analyzed current amplitudes in control (Figure 6F, red bar) and in the presence of 1 mM IBMX (Figure 6F, green bar) either in 2 mM (filled bars) or 0-added extracellular calcium (striped bars) and found that IBMX induced a significant current increase in both conditions. The mean value of the net IBMX-induced current in 2 mM Ca^2+^ (3.67±1.37 pA/pF) was not significantly different from that in 0-added Ca^2+^ (3.23±1.03 pA/pF) or from that induced by 8-Br-cGMP (3.50±0.85 pA/pF) in 2 mM Ca^2+^ (data not shown). The relation between basal current amplitudes and net currents distributions for D30 cells in 2 mM Ca^2+^ was very similar for IBMX and 8-Br-cGMP (Figure 6G), except for the fact that IBMX was not tested on RNS-derived cells having basal inward currents larger than −10 pA/pF. In control experiments, no response to IBMX was recorded from cells recorded in whole-cell mode with an intracellular solution lacking ATP and GTP to block cGMP synthesis by the cyclase, suggesting that IBMX does not open the channels in the absence of cGMP synthesis (data not shown).

To gain further insight into cGMP turnover, we evaluated the effect of changes in Pde activity on endogenous cGMP accumulation. A dose-dependent increase in cGMP accumulation by cells incubated in the presence of the Pde inhibitor IBMX [20] suggested that endogenous cGMP was controlled through Pde (Figure 6H). The observation that IBMX application increased calcium accumulation (Figure 6I) suggested that the increase in endogenous cGMP induced by IBMX translated into an increased activation of cGMP-gated channels.

### Responses to light of RNS-derived rods

**Figure 7:**
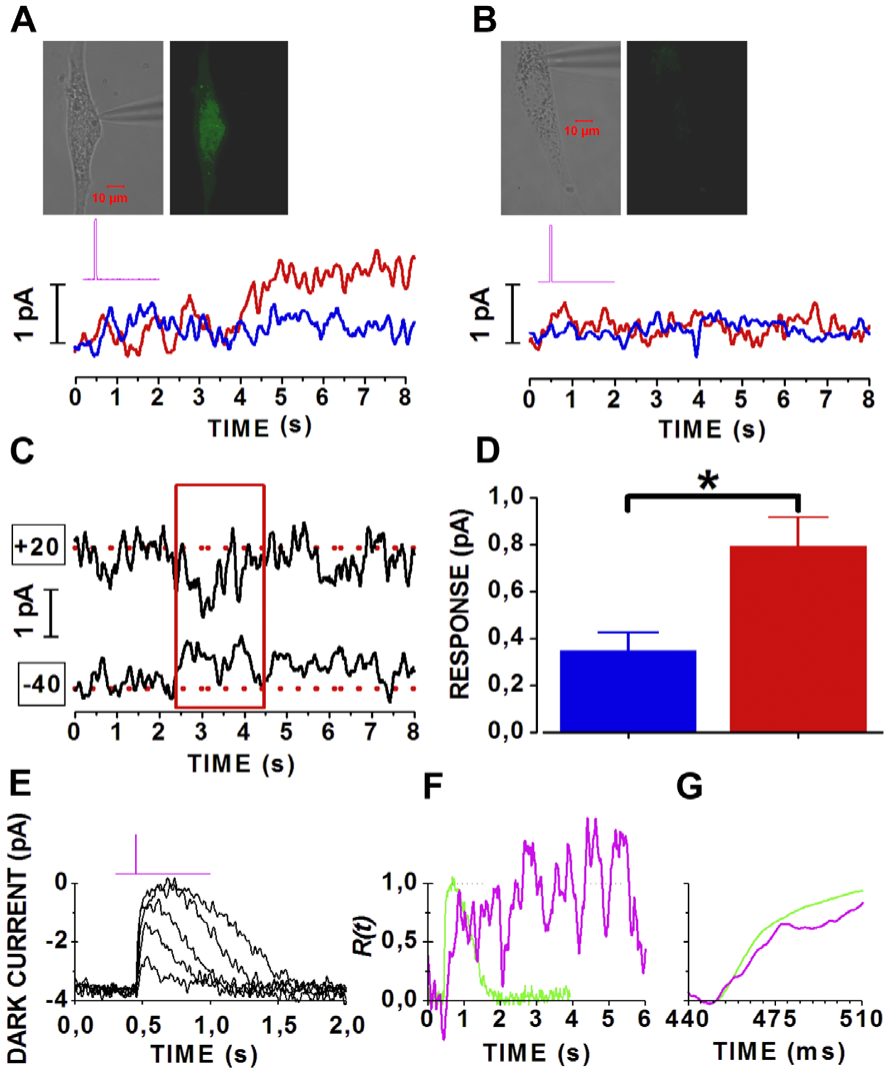
Light response of RNS-derived rods. (**A**) Responses to flashes delivering 719,500 (red) and 144,600 (blue) photons µm^−2^, respectively. Each trace is the average of 25 individual sweeps, each one recorded in response to a 50 ms-long flash applied every 30 s. The recorded 45 days-old cell is shown above records. The magenta line plots the time course of the light flash. (**B**) Average currents recorded from an EGFP^−^ cell (shown above sweeps) using the same experimental conditions as the cell in A. (**C**) Average responses to flashes delivering 356,100 photons µm^−2^, recorded at holding voltages of −40 and +20 mV. The horizontal dashed red lines plot the baseline levels of −56.7 and +74.8 pA at the holding potentials of −40 and +20 mV (indicated by black boxes on the left of records), respectively. Responses of different polarities are enclosed by the red box. In A, B and C experimental records were smoothed by a 100-points window to further reduce noise. (**D**) Average response amplitudes recorded from 5 cells in response to flashes delivering 719,500 (red) and 144,600 (blue) photons µm^−2^. *, p<0.05 by paired t-test. (**E**) Response of an adult mouse rod to a 2-ms long flash delivering 7, 18, 52, 153, 415 and 1,015 photons µm^−2^. Each trace was the average of 9 individual sweeps. The magenta line plots stimulus time course. (**F**) Fractional responses of an adult rod (green) and a RNS-derived cell (magenta) to saturating light stimuli delivering 719,500 and 1,015 photons µm^−2^, respectively. Traces were the average of 9 and 5 responses for the adult rod and the RNS-derived cell, respectively. (**G**) Fractional responses from **F** plotted on an expanded time scale. Temperature differences were compensated by a Q10 of 3.5.

We next investigated whether *in vitro*-generated rods express the entire phototransduction machinery and are therefore able to modulate endogenous cGMP through the biochemical pathway triggered upon photon absorption by Rho. Cells differentiated *in vitro* were grown in the absence of pigment epithelial cells converting all-*trans* retinal to 11-*cis* retinal, necessary for Rho regeneration from rod opsin. To allow RNS-derived rods generating a functional Rho, we incubated cells with 9-*cis* retinal (see Methods), a commercially-available homologue of the natural chromophore 11-*cis* retinal [21]. Cells were visualized under infrared light and EGFP expression was evaluated after recording, to avoid regenerated Rho being fully bleached by the EGFP excitation light (see Methods). In EGFP^+^ cells the highest intensity (red trace in Figure 7A) evoked a saturating response of larger amplitude than that evoked by the dimmer stimulus (blue trace in Figure 7A). Light stimuli did not evoke responses from EGFP^−^ cells (Figure 7B), suggesting that light responsiveness is a specific feature of Rho expressing cells. In order to assess whether these responses were consistent with the closure of cGMP-gated channels, we measured their polarities at two different holding potentials. Light stimuli reduced the inward holding current (−56.7 pA) at −40 mV and the outward holding current (+74.8 pA) at +20 mV (red box in Figure 7C), consistent with cGMP-gated currents reversing close to −10 mV (see Figure 5G and 6E). Flashes delivering about 150,000 photons µm^−2^ closed about half of the channels open in darkness (Figure 7D). This intensity is nearly a 1000-fold higher than that required for halving the dark current of adult mouse rods, as shown in Figure 7E for an adult mouse rod, indicating that RNS-derived rods have low light-sensitivity. Light responses of RNS-derived cells had longer latencies (up to 3 s) than those of adult rods (10–15 ms). Sweeps in Figure 7F plot normalized responses recorded from an adult rod (green) and an RNS-derived cell (magenta), respectively, after a temporal shift to align the foot of the response of the RNS-derived cells to that of the adult rod. To compare their time course, the fractional response [22] computed from the data in 7F was plotted in Figure 7G after compensating temperature difference (22°C for the RNS-derived cell and 36°C for the adult rod) by a Q10 of 3.5 [23]. These results suggest that RNS-derived EGFP^+^ cells generate some rudimentary light responses but their small amplitudes and variable latencies prevented further investigation.

### Functional maturation of RNS-derived rods

**Figure 8:**
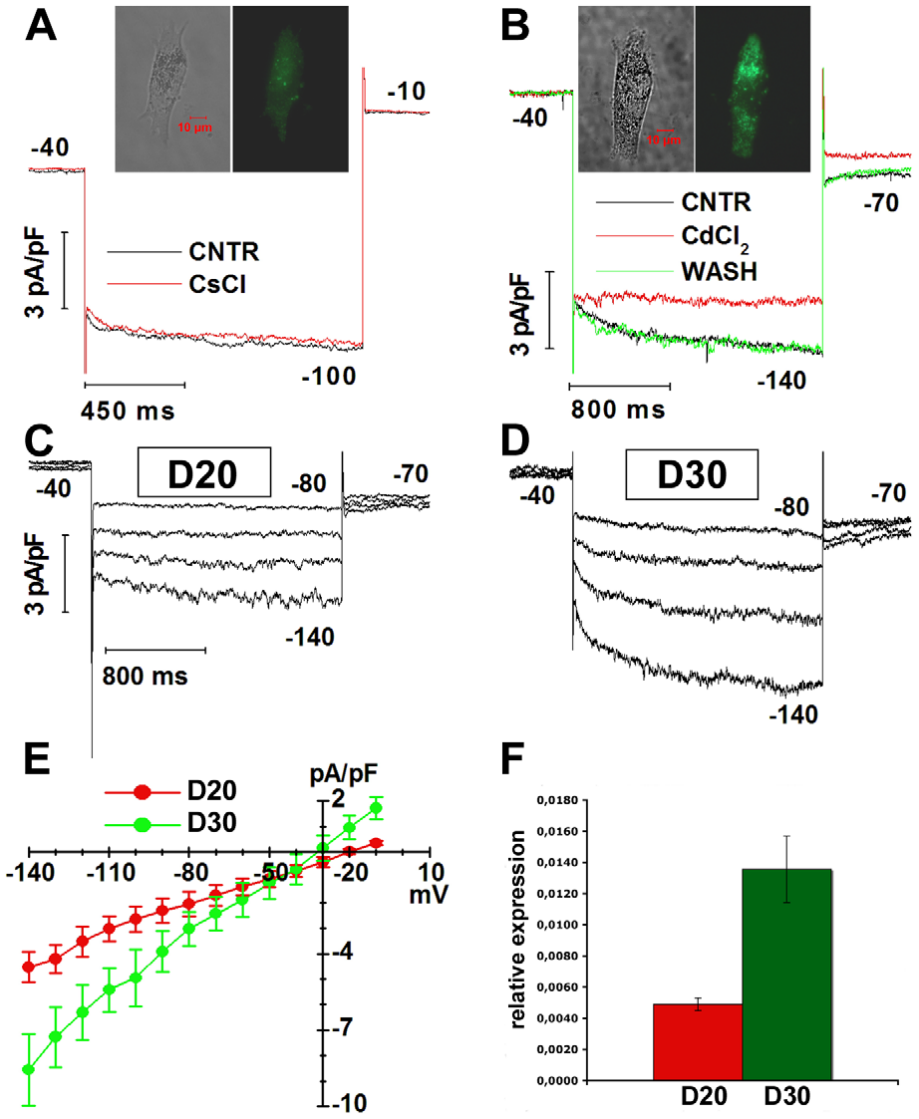
Hyperpolarization-activated currents through Clc-2 channels in RNS-derived rods. (**A**) Traces plot normalized inward currents generated by an EGFP^+^ cell (images of the cell are shown above) in response to a hyperpolarizing voltage step from −40 to −100 mV either in Locke solution (CNTR – black trace) or in the presence of 3 mM CsCl (CsCl - red trace). Step duration was 1.2 s. Voltages are indicated close to the traces. (**B**) Traces plot normalized inward currents activated in response to a hyperpolarizing voltage step from −40 to −140 mV in Locke solution (CNTR – black trace), in the presence of 2 mM CdCl_2_ (red trace) and after reverting to Locke solution (WASH, green trace). Step duration was 2 sec. Images of the recorded cell are shown above traces. (**C–D**) Sweeps plot normalized currents activated by membrane hyperpolarizations in EGFP^+^ RNS-derived cells at D20 (C) and D30 (D). Voltage steps ranged from −80 to −140 mV in 20 mV-steps, from a holding of −40 mV. Numbers close to the experimental records in A–D indicate the applied voltage. Vertical deflections in A–D are capacitance transients elicited by voltage changes. Calibration bars hold for both C and D. (**E**) Data points plot mean normalized currents, with their s.e.m., as a function of activating voltages, for D20 (red, N = 8) and D30 (green, N = 10) RNS-derived cells. For each cell the last 200 ms of the 2 s-long steps were averaged. (**F**) Real-time PCR evaluation of *Clcn2* expression in RNS-derived cells at D20 (red), and D30 (green). Bars plot s.e.m. from a single experiment performed in triplicate. Data derive from the formula 2^−ΔCt^.

We investigated the expression of voltage-gated currents by rod-like cells to compare their electrophysiological signature with those of adult rods and ciliary epithelial cells. Similar to adult rods, but in contrast to CE cells [24], EGFP^+^ RNS-derived cells generated a slowly-activating inward rectifying current in response to membrane hyperpolarization (Figure 8A, black trace). CsCl, a blocker of Hcn1 and Kcnj14 channels that carry inward rectifying currents in adult rods [24], failed to block the inward current of the RNS-derived cells (Figure 8A, red trace). In a second cell, a complete and reversible block of the inward rectification was observed with 2 mM CdCl_2_ (Figure 8B, compare black, green and red traces), as expected for Clc-2 chloride channels encoded by the *Clcn2* gene. Normalized amplitudes of hyperpolarization-activated currents increased with maturation, as shown for two representative RNS-derived cells at D20 (Figure 8C) and D30 (Figure 8D). The increase in average normalized current amplitudes shown in Figure 8E was consistent with *Clcn2* mRNA expression being higher at D30 than at D20 (Figure 8F).

**Figure 9:**
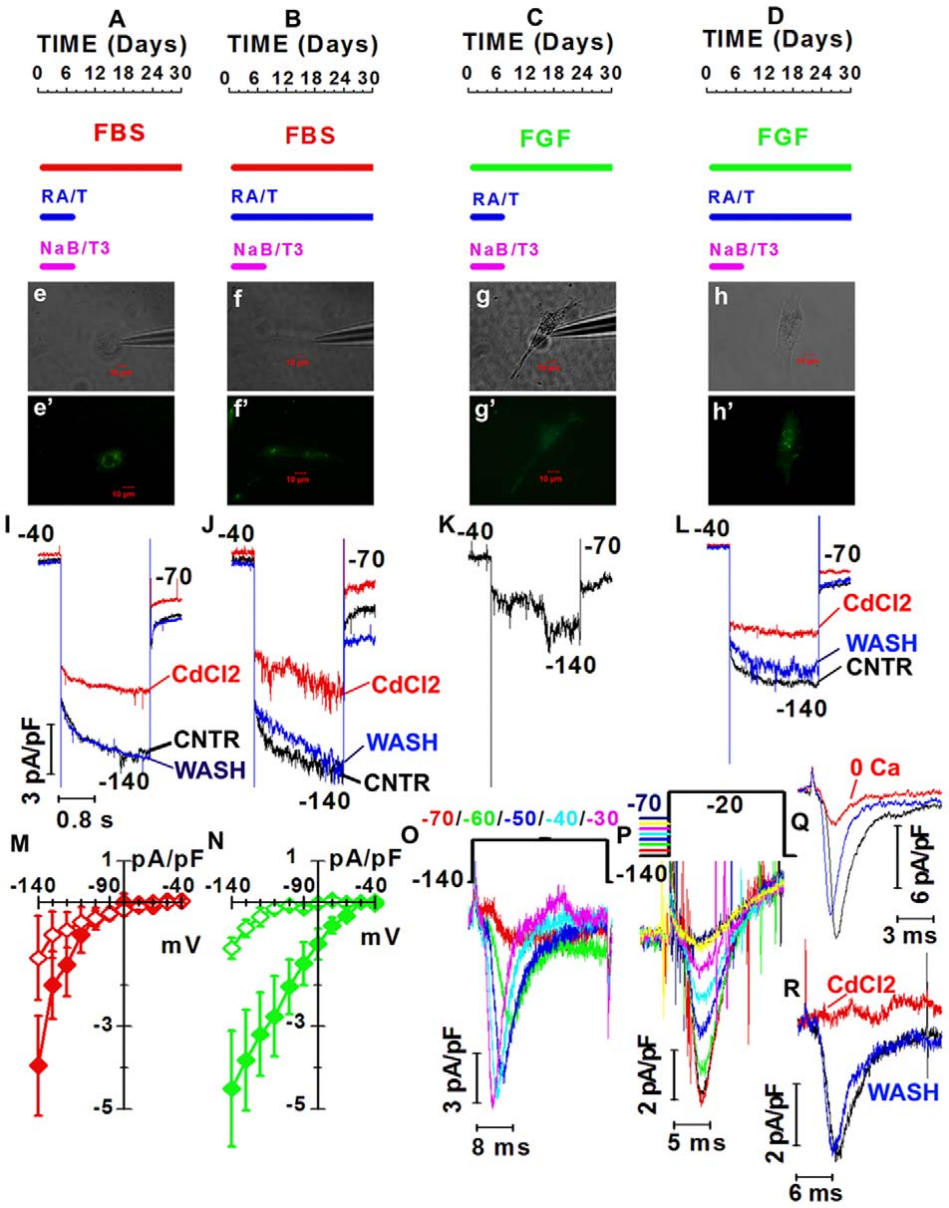
Effects of differentiation media on the expression of voltage-gated currents by rod-like cells derive from RNS. (**A–D**) Red, green and magenta bars: time of exposure to fetal bovine serum (FBS), basic fibroblast growth factor (FGF) and sodium butyrate (NaB) and T3, respectively. Blue bars: exposure time to retinoic acid (RA) and taurine (T) for 7 (A and C) and 30 days (B and D). Time 0: beginning of treatment and corresponds to the 5^th^ day in culture (D5). (**e–h′**) B/W and fluorescent images of cells cultured as shown in A (e, e′), B (f, f′), C (g, g′) and D (h, h′). (**I–L**) Normalized hyperpolarization-activated currents before (black – CNTR), during (red – CdCl_2_) and after washing out 2 mM CdCl_2_ (blue – WASH) by cells shown in e (I), f (J), g (K), h (L). Calibration bars in I also hold for panels J–L. (**M–N**) Net current densities recorded in either FBS (M, red diamonds) or FGF (N, green diamonds) with NaB/T3 plus RA/T either for 7 (red open diamonds, N = 5; green open diamonds, N = 7) or 30 (red filled diamonds, N = 4; green filled diamonds, N = 10) days. (**O**) Depolarization-activated fast inward currents in a D30 cells cultured in the presence of FGF and 1week treatment with RA/T/NaB/T3 (panel C). Black trace: time course of voltage steps. Color-coded numbers: voltages match noisy records. (**P**) Inward currents evoked by a 20 ms-long step from −140 to −20 mV were rapidly inactivated. The pre-pulse voltages are plotted by the black line above the records. (**Q**) Inward currents in a cell cultured as in panel A, before (black trace), during (red trace – 0 Ca) and after exposure to saline with 0-added Ca^2+^ (blue trace). (**R**) Inward currents of a cell cultured as in A before (black trace), during (red trace – CdCl_2_) and after exposure to 2 mM CdCl_2_ (blue trace - WASH).

Clc-2 current up-regulation required continuous exposure to RA and Taurine (Figure 9). Of note, most cells treated for only 1 week with RA and taurine initially activated Rho, as shown by EGFP expression (Figure 9 panels e′–f′, g′–h′), but failed later on to up-regulate Clc-2 currents (open diamonds in Figure 9 M–N) and instead expressed fast inward calcium currents, similar to rod bipolar [25] rather than to rod cells (Figure 9 O–R).

## Discussion

In this study we provide evidence that functional rod-like photoreceptors can be obtained *in vitro* from RNS derived from the adult CE. Our data confute two previous publications suggesting that RNS have CE characteristics and that they fail to generate cells expressing Rho [9], [10]. In sharp contrast, we confirmed expression of Rho with two monoclonal antibodies. This ruled out the possibility that we were analyzing “false rods” labeled by nonspecific immunofluorescence of distorted edges of dead cells as suggested by [9], [10]. We need to point out that culture conditions in previous studies highly diverge from the protocol here described. Second, we showed functionality of the *Rho* gene promoter after infection with an AAV2/8 system. We also confuted the claim that RNS are CE cells, rather than stem cells, by showing that RNS expressed *Nanog* and, at higher levels compared to ES cells and CE, retinal progenitor genes such as *Pax6*, *Chx10*, *Nestin* and *Rax*. Although the CE expressed components of the phototransduction cascade [26], [27], we found lower levels of *Rho*, *Crx*, *Nrl* and *Nr2e3* in RNS compared to the CE. These data suggest that RNS derived from pigmented cells of the CE but diverged from the CE, and support the hypothesis that RNS cells have retinal progenitor characteristics, in strong contrast to the hypothesis of transdifferentiation of CE-like cells into photoreceptors proposed by [9], [10]. Consistent with this notion, RNS-derived rods differentiated from retinal degeneration (*rd1*) mutant mice or bearing a rhodopsin mutation, undergo spontaneous cell death *in vitro* [28], [29] corroborating the idea that the differentiation procedure does not reconvert RNS cells into CE cells, because CE is not affected by this disease. Our data are in line with the previous study on CE derived RNS suggesting that cells in RNS have a mixed neural and epithelial characteristics and *in vitro* culture conditions can regulate the balance between epithelial and neural properties [30].

The conclusion that RNS derived from the CE are able to generate rod-like cells, is based on both molecular and functional evidence, a novel approach compared to those previously adopted in the field, often relying on the expression of few genes and/or proteins to infer about fate assignment and functionality. First, we analyzed the co-expression of Rho with several proteins involved in phototransduction. Furthermore, using the AAV2/8-pRho-EGFP construct to compare at the single-cell level the maturation of rod-fated cells with those that had not activated the rod differentiation program, we characterized macroscopic currents through rod-specific channels demonstrating that only EGFP^+^ cells responded to cGMP by activating a current whose biophysical properties of low noise (i.e. small unitary conductance), reversal close to −10 mV and its sensitivity to the calcium electrochemical gradient (i.e. mixed cationic permeability) and lack of voltage-dependence were consistent with cations permeation through cGMP-gated channels, as confirmed by calcium imaging. These functional features correlated with *Cnga1* expression by RNS-derived cells. In our studies we estimated single channel amplitude of 0.1–0.2- pA in the presence of divalent cations that translated to a conductance of about 0.75–1.5 pS, consistent with gating of channels of low unitary amplitude by 8-Br-cGMP. These data do not necessarily diverge from the lower value of 0.1 pS estimated by by Fesenko [19], because their use of mM concentrations of divalent cations also on the intracellular side, can reduce the cGMP-gated channel conductance [31]. The nearly 20 mV difference between reversal potentials in 2 mM and 0-added Ca^2+^, of both 8-Br-cGMP and IBMX-induced currents was also larger than that reported for native rod cGMP-gated currents [32] and more similar to that reported for cone channels [32]. This observation opens the possibility that RNS-derived cells may also express additional Cnga isoforms and more work is required to specifically address the relative contribution of different Cnga isoforms to the cGMP-gated current of RNS-derived cells.

It must be pointed out that uncertainties about the relative contribution of different Cnga isoforms to cGMP-induced currents do not change the main conclusions of this work, that RNS-derived cells have the ability to synthesize cGMP, allowing endogenous cGMP to gate a fraction of calcium permeable cGMP channels. Patch-clamp and calcium imaging data provided evidence that an increased number of cGMP-gated channels were activated by endogenous cGMP, a finding consistent with increased expression during *in vitro* maturation of *Cnga1*, as well of *Gucy2f* and *Gucy1a*, coding for the guanylate cyclase and its activator, respectively. The increase in cGMP content induced by IBMX and the activation of a current with properties similar to that activated by 8-Br-cGMP suggested that Pde6 was active as well. IBMX also caused an increased calcium accumulation, a result consistent with the activation of cGMP-gated channels by endogenously-generated cGMP. Of note, calcium accumulation by IBMX-treated cells was similar to that of cells derived from *rd1* mice (not shown), which have a genetic defect causing a nearly complete loss of Pde activity.

We also analyzed the ability of rod-like cells to generate light responses. Although RNS-derived cells could hardly be considered functional photoreceptors because amplitudes of light-induced responses were low, using averaging to reduce noise we measured small-amplitude responses whose polarities were consistent with the suppression of currents through cGMP-gated channels by light stimuli. The low sensitivity to light, which is over a 1000-fold lower in rod-like cells compared to adult rods, and the long latency between stimulus and response suggest that rod-like cells express all the components required for phototransduction, although in the absence of the spatial organization provided by the outer segment the chance of an encounter between these proteins is quite low. It is relevant to note that the generation by RNS-derived rods of light responses of smaller amplitude and lower sensitivity than those of adult mouse rods is reminiscent of P10 (post-natal day 10) rats and developing rods of *Xenopus* tadpoles, whose light responses are of smaller amplitude and lower sensitivity than those of adult animals [21], [33]. Additional evidence of immaturity of RNS-derived rods comes from patch-clamp recordings of voltage-gated currents. Although both adult rods and RNS-derived rod-like cells express inward-rectifying currents, they were carried through Hcn1 and Kcnj14 (Kir2.4) channels in adult rods and through Clc-2 channels in RNS-derived cells. The finding that photoreceptors of *Clcn2* knock-out mice lack normal outer segments and degenerate between P10 and P30 [34], suggests that Clc-2 channels play a transient role during rod maturation that is critical for viability, but more work is required to specifically address this point by recording from immature rods to evaluate the transient expression of Clc-2 channels.

It is interesting to note that the up-regulation of *Clcn2* expression is not an unspecific cell derangement caused by long culture time, as it requires prolonged treatment with RA and taurine. Cells treated for 1 week-only with RA and taurine did activate Rho expression, but failed to up-regulate Clcn-2 expression and rather express fast inward calcium channels. Similar inward calcium currents are expressed by rod-bipolar cells [25], a different late-born retinal neuron, but not from CE cells, consistent with the notion that RNS-derived cells have retinal rather than CE characteristics. It seems that stem cells in the CE are plastic and are able to generate both RPE and retinal neurons depending on the culture conditions applied [8], [35]. It must be noted that our differentiation protocol using RA and Taurine for 30 days highly diverges from those reported in studies in which rod markers could not be detected [9], [10].

Development of therapeutic approaches for retinal degeneration is hampered by the lack of *in vitro* models suitable for high-throughput screens of drugs. Several animal models are available but are not appropriate for systematic studies. Generation of rod photoreceptors *in vitro* can open new approaches for testing therapies for retinal dystrophies. Here we provide a differentiation protocol to obtain high percentages of cells with rod characteristics. Furthermore, we previously showed that RNS-derived cells bearing the P347S rhodopsin mutation or a loss of function of the *Pde6b* gene could be used to select pharmacological and molecular tools for therapy approaches [28], [29].

Dedicated studies will be necessary to define if these cells have also the potential to produce cells amenable for rod cell replacement. MacLaren and colleagues reported that precursors from P4-P5 mice integrate better than cells from embryonic or adult retinas when transplanted into the subretinal space of adult mice [36], suggesting that rod transplantation requires immature post-mitotic rod precursors. Murine rod-precursors at P5 are post-mitotic, are going to turn on Rho expression and they have not developed an outer segment. Similarly, the cells that we obtain *in vitro* are post-mitotic, have turned on Rho but they are still morphologically and functionally immature. Consistent with the notion that RNS-derived rods are immature and amenable to transplantation, cells derived from RNS had been previously shown to integrate and develop an outer segment upon transplantation in neonatal mice [37].

## Methods

### Retinal stem cell isolation and differentiation

All procedures on mice were performed in accordance with the ARVO Statement for the Use of Animals in Ophthalmic and Vision Research and with institutional guidelines for animal research. We used 3–5 months-old C57BL/6 mice and RNS were clonally generated as previously reported [8]. Primary RNS were seeded on glass coverslips coated with extracellular matrix (ECM, Sigma). Cells were cultured in serum free media (SFM) supplemented with 20 ng/ml bFGF and 2 µg/ml heparin. The cells were allowed to proliferate and migrate out of the sphere over the course of 4 days. The medium was replaced at the 5^th^ day (D5) with medium supplemented with 10 ng/ml bFGF, 2 µg/ml heparin, 500 µM taurine, 2 mM sodium butyrate (NaB), 100 nM RA and 5 nM triiodothyronine (T3). After 7 days (D12) NaB and T3 were removed because we found that NaB at this dose can be toxic for longer times, as previously reported for retinoblastoma cells [12]. A schematic representation of the differentiation protocol can be found in Figure 9A–D. Cells were infected at D4 with an AAV2/8 expressing EGFP under the control of the rhodopsin promoter (AAV2/8-pRho-EGFP) (1×10^6^ GC/cell) according to the procedure described in [15].

### Cell proliferation and cell death evaluation

Cell at D4, D6 and D8 were treated with 10 µM bromodeoxyuridine (BrdU) for 3 h, then proliferating cells were detected as previously described [38].

Cell death was assessed by staining for 1 minute with 2 µM of the cell impermeable dye Ethidium Homodimer 2 (Invitrogen).

### Real-time PCR

Total RNA was extracted from RNS and *in vitro* differentiated RNS-derived rods using RNeasy Mini Kit (Qiagen) according to the manufacturer's instructions. cDNA was synthesized using Transcriptor High Fidelity cDNA Synthesis Kit (Roche). Real-time PCR was carried out with the ABI PRISM 7900 HT Sequence Detection System (Applied Biosystems) as previously described [8] and normalized using the ribosomal gene *S26*. The primers used for quantitative PCR are listed in Table 1 and PCR products were checked by sequencing.

**Table 1:** Sequences of primers used in Real-time PCR experiments.

GENE	PRIMER SEQUENCE (5′- 3′)
***Chx10 (Vsx2)***	F: TCCGATTCCGAAGATGTTTC
	R: GTGGGCTTCATTGAATGCTT
***Clcn2***	F: CTGCCCTACCTACCTGAGC
	R: AAAGTGCAGCTGAGGGCTAC
***Cnga1***	F: CAACTGGACGATGATTATTGC
	R: TCACTAGCAGCCCTTGTTCC
***Gnat (Transducin)***	F: GAGCCTCAGAATACCAGCTC
	R: GGCACATATCCTGGAGTCAC
***Guca1a***	F:TGCATAGACAGGGACGAGC
	R:GCACTCATGGATGAGTCGC
***Gucy2f***	F:TACGAAGCCTATGCAGAAGC
	R:ATTGTAGATGGTTCCAAACAAC
***Mitf***	F: TCTGAAGCAAGAGCATTGGCT
	R: AGCTCCTTAATGCGGTCGTTT
***Nestin***	F: AGCTGGCTGTGGAAGCCC
	R: CTCTGTAGACCCTGCTTCTC
***Pax6***	F: GCGGAAGCTGCAAAGAAATA
	R: TGGCCTGTCTTCTCTGGTTC
***Pde6b***	F: GGAGAGGACTGTCTTGGATC
	R: GAGCTCAGCTGCTTTGTTCC
***Rax***	F: GGAACCTCCCAAGAAGAAGC
	R: GCTGTACACGTCGGGGTAGT
***Rhodopsin (Rho)***	F: AATCTCGAGGGCTTCTTTGC
	R: CCACGTAGCGCTCAATGGC
***Rlbp1 (Cralbp)***	F: TCTTGAAGAACAAGCTGCTAC
	R: CTGTGTTCTCAACTTCAGCC
***S26***	F: AAGTTTGTCATTCGGAACATT
	R: GATCGATTCCTAACAACCTTG

### Immunofluorescence

Immunofluorescence experiments were performed as previously reported [8]. Primary antibodies were used as follows: 1∶100 anti-rhodopsin mouse monoclonal 1D4 (Sigma-Aldrich), 1∶1000 anti-rhodopsin mouse monoclonal RET-P1 (Sigma-Aldrich), 1∶100 anti-Pde6b rabbit polyclonal (ABCAM, Cambridge, UK), 1∶100 anti-recoverin rabbit polyclonal (Millipore), 1∶100 anti-peripherin rabbit polyclonal (Millipore), 1∶300 anti-transducin rabbit polyclonal (Sigma-Aldrich), 1∶100 anti-AIF rabbit polyclonal (Sigma-Aldrich). Secondary antibodies were as follows: 1∶1000 Oregon Green® 488 goat anti-mouse (Molecular Probes) and 1∶1000 Alexa Fluor® 568 goat anti-rabbit (Molecular Probes). Coverslips were mounted with mowiol 4–88 (Sigma-Aldrich) and analyzed at a Zeiss Axioskop 40 fluorescence microscope.

### Calcium imaging

RNS-derived cells were pre-treated with 0.5–2 mM Iso-Butyl-methylxanthine (IBMX) or 20 µM 8-Br-cGMP. Fluo-4 AM (Molecular Probes) was loaded into cells for 30 min at 37°C in calcium free medium. Cells were excited using a 488 nm laser beam and analyzed with a Leica Laser Confocal Microscope System. 10 sequential layers of each cell were photographed. Images were then analyzed with the Leica confocal software (LCS). The fluorescence average intensity of each area was measured.

### cGMP measurement

cGMP concentration in D30 RNS-derived cells was evaluated after treatment with IBMX for 30 min with the Amersham cGMP Enzymeimmunoassay Biotrak EIA System (GE Healthcare) following the manufacturer's instruction.

### Patch-clamp

A coverslip with RNS-derived cells was transferred to a custom-made recording chamber, mounted on the stage of a Leica DMI 4000B inverted microscope and continuously superfused with Locke solution [24] at a rate of 0.5 ml/min, requiring about 2–4 minutes for equilibration with the 2–3 ml bath volume. Cells were visualized through a 60× objective and images acquired using a Leica (DFC-350 FX) digital camera. When investigating the expression of voltage-gated or cGMP-gated channels, cells were selected on the basis of EGFP fluorescence.

Mean gray values from fluorescence images acquired using a constant exposure time and light intensity were computed using Image J 1.43 freeware (McMaster Biophotonics, USA), binned and their distribution fitted by a Gaussian function (Origin 6 from Microcal, USA). The mean gray values distribution was well fitted by a single Gaussian function, and cells were classified as high or low fluorescence when their mean gray value were outside +1 or −1 SD, respectively. We compared cells from the same differentiation experiment. For instance, cells in Figure 5 (A and C) and 7 (A and B) were recorded the same day, making the comparison straightforward. For figure 9, most cells were acquired within a 2-months time-span and the fluorescence distribution was not altered during this period.

For recording of light responses, RNS-derived cells were incubated for 12–36 hours at 37°C in darkness with 200–500 nM 9-*cis* retinal, a commercially available homologue of 11-*cis* retinal (Sigma-Aldrich). RNS-derived cells were visualized using infrared light, by inserting a long-pass filter (λ>800 nm) into the light path as previously reported for *Xenopus* tadpoles [39], and EGFP fluorescence was monitored at the end of recording, to avoid rhodopsin bleaching by the high-intensity EGFP excitation light (470–490 nm). An ultrabright LED (OD-520, Optodiode) [40], mounted on the microscope second camera port, was the stimulus light source. Using the 60× objective, light was focused on cells as a circular spot of 1.2 mm diameter, and its energy measured through an optical power meter (Newport model 1815-C) and converted to photons µm^−2^ flash^−1^. Light stimuli were 50 ms-long flashes, applied every 30 seconds to let cells recover completely after illumination.

For measuring light responses from adult mouse rods, mice were sacrificed using a lethal dose of anesthetic (2 ml of 20% urethane), and retinas quickly isolated through a corneal slit as previously reported [41]. Responses were measured in Locke's solution using the suction pipette technique, as previously reported for adult monkey and guinea pig [42].

Cells were recorded using the perforated-patch technique, and data acquisition and stimulation were as previously reported for adult mouse rods [24]. Membrane capacitance and resistance as well as access resistance were regularly monitored. For 8-Br-cGMP and IBMX-gated currents recordings the voltage was ramped from −80 to +50 mV at a rate of 0.542 V s^−1^ and currents digitized at 33 kHz after low-pass filtering at 3 kHz. Effects of 8-Br-cGMP and IBMX on membrane current were measured at a holding voltage of −80 mV. For monitoring of voltage-gated currents we used voltage steps as previously reported in adult mouse rods [24]. Hyperpolarization-activated currents were evoked by 2 s-long voltage steps ranging from −40 to −140 mV in 10-mV steps from a holding of −40 mV. Net current amplitudes were computed by subtracting average current amplitudes measured in the interval 575–600 ms from those in the interval 2450–2550 ms.

Inward calcium currents were activated by 20–30 ms long voltage steps to potentials ranging from −70 to −20 mV, from a holding of −140 mV. For investigating current inactivation the activation step was preceded by a 50 ms-long pre-pulse at voltages ranging from −140 to −70 mV in 10 mV steps. Currents were sampled at 50 kHz after filtering at 5 kHz and capacitive and resistive components subtracted on-line by a P/5 protocol.

### Data analysis

Unitary current amplitude through cGMP-gated channels was estimated by spectral analysis of macroscopic current. Briefly, 3 to 5 recording stretches 25 ms-long acquired at a holding voltage of −80 mV, either before or during exposure to 8-Br-cGMP, were used for computing current variance (σ*_i_*) according to Eq. 1:
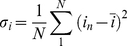
(1)where *N* is the total number of points in the sweep (*N* = 825), *i_n_* is current value of point *n* and *i* is the average current value.

The variance increase due to 8-Br-cGMP was estimated by subtracting current variance before the application of the analogue from current variance in the presence of 8-Br-cGMP. The elementary current was estimated by dividing the variance increase by the 8-Br-cGMP-induced current *I*, according to equation 2:
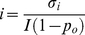
(2)where *p_o_* is the open channel probability, which according to Matthews and Watanabe [43] we took equal to 0.3.

Power spectrum analysis of 8-Br-cGMP-induced current noise was carried out using Clampfit (Axon Instruments, CA), using 512 points from four 25 ms-long recording stretches acquired at 15 s intervals at an holding of −80 mV, either before or during exposure to the cGMP analogue. Spectral data were then smoothed by a 5 points window and fitted by a Lorentzian function:
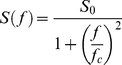
(3)where *f* is the frequency, *S_0_* is the extrapolated spectral component for frequency 0 and *f_c_* is the frequency at which *S(f)* = *S_0_*/2.

The time constant ***τ*** was computed by
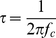
(4)
***τ*** provides an approximation to the average channel opening lifetime.

All data are shown as mean ± s.e.m. Independent and paired t-test were carried out using Origin 5.0 (Microcal). One-way and two-way ANOVA, with Tukey's tests for multiple comparisons were carried out using Origin 8.5 (Microcal).
