# Correction: Functional and Molecular Characterization of Rod-like Cells from Retinal Stem Cells Derived from the Adult Ciliary Epithelium

**DOI:** 10.1371/annotation/ca21f359-8e8e-4c3d-8308-e0f20fc446bb

**Published:** 2012-08-10

**Authors:** Gian Carlo Demontis, Claudia Aruta, Antonella Comitato, Anna De Marzo, Valeria Marigo

There was an error in the published funding statement. The correct funding statement is:

V.M. was supported by research grants GGP06096 from Fondazione Telethon, research grants 2006053302_003 and 20094CZ3M2 from the Italian Ministry, Progetti di ricerca a carattere internazionale from Fondazione Cassa di Risparmio di Modena and by the European Union (grant n. LSHG-CT-2005-512036). G.C.D. was supported by research grant 2006053302_002 and 20094CZ3M2_003 from the Italian Ministry of Education and by Fondazione ARPA. The funders had no role in study design, data collection and analysis, decision to publish, or preparation of the manuscript.

